# Trends in Multicomponent Training Research in the Aged Population: A Bibliometric Analysis

**DOI:** 10.3390/healthcare12151493

**Published:** 2024-07-27

**Authors:** Damián Pereira-Payo, Ángel Denche-Zamorano, María Mendoza-Muñoz, Juan Manuel Franco-García, Jorge Carlos-Vivas, Jorge Pérez-Gómez

**Affiliations:** 1 Health, Economy, Motricity and Education (HEME) Research Group, Faculty of Sport Sciences, University of Extremadura, 10003 Cáceres, Spain; dpereirapayo@unex.es (D.P.-P.);; 2 Promoting a Healthy Society Research Group (PHeSO), Faculty of Sport Sciences, University of Extremadura, 10003 Cáceres, Spain; 3 Physical and Health Literacy and Health-Related Quality of Life (PHYQoL), Faculty of Sport Science, University of Extremadura, 10003 Cáceres, Spain; 4 Physical Activity for Education, Performance and Health (PAEPH) Research Group, Faculty of Sport Sciences, University of Extremadura, 10003 Cáceres, Spain

**Keywords:** multicomponent exercise, older adults, physical functional performance, scientometrics

## Abstract

The proportion of aged populations is increasing worldwide. Exercise has a palliating effect on some adverse implications of aging. Multicomponent training (MCT) is a recommended form of exercise for the aged population. The aims of this research were to (1) study the number of publications regarding MCT in the aged population following an exponential growth rate; (2) identify the journals, authors, and countries that stand out the most in this area; and (3) describe the most common themes and used keywords in this field. The analysis was performed through the traditional laws of bibliometrics, including, Price’s, Lotka’s, Bradford’s, and Zipf’s law. All documents published in journals indexed in the Web of Science (WoS) Core Collection from 2001 to November 2023 that met the inclusion criteria were included. The 485 documents included in this review revealed that the number of annual publications experienced an exponential growth phase, 15 journals with six or more publications formed the core journals on this topic, and the author Mikel Izquierdo and his collaborative network topped the lists of prominent and prolific co-authors. Spain was the leading country in number of publications. Various thematic lines and keywords regarding strength, sarcopenia, quality of life, falls, balance, dual-task exercise, and cognitive and physical functioning were identified. In conclusion, this work confirmed that research on this topic is going through an exponential growth phase and provided detailed information about the journals, authors, and countries involved in the subject, as well as the keywords most frequently used in the subject matter.

## 1. Introduction

The global population is rapidly aging, and the percentage of people aged 60 or above is around one-third of the total population in some countries [1]. The aging process is associated with a series of physiological changes that lead to a concomitant increase in the risk of chronic disease and multimorbidity [2]. From the age of 60 years onwards, there is a decline in the senses and a loss in mental and physical functioning that increases the susceptibility to disability and the odds of frailty [2,3]. In this sense, performing regular physical activity (PA) has been shown to be an important factor in the prevention of physical decline and in slowing the adverse effects of aging in the elderly population [4,5]. Thus, a type of exercise that integrates the development of various physical capacities in the same program could be particularly beneficial. This makes multicomponent training (MCT) one of the preferred forms of exercise for the aged population since it has been proven effective in improving various physical capacities (agility, upper limb strength, lower limb strength, and aerobic capacity [6]) and maintaining and improving the autonomy and the quality of life of older adults [7].

MCT has been defined as the combination of three or more training types within the same training program [6]. MCT brings together various physical training modalities, each one specific for the development of one or more physical capacities. It is suggested that MCT programs should be composed of a combination of endurance, strength, coordination, balance, and flexibility exercises [8]. However, in some cases, in the context of the aged population, some MCT proposals only include the work of some of these physical qualities to develop and maintain the functional capacity of older practitioners. Strength [9,10] and endurance [10,11] training are usually present in these exercise programs, accompanied by the work of other physical qualities. Occasionally, activities with a more playful and collective character are included, such as dance or music-based exercises that are used in the framework of balance or gait ability, which, in addition to fulfilling the main objective of developing a particular physical quality, are also effective in improving the intrinsic motivation for exercise [12,13].

The combination of various exercise types within an MCT program has shown its effectiveness in improving a series of physical, cognitive, and biochemical parameters [14,15,16,17]. MCT has been found to be effective in improving physical condition [6], gait ability [18], balance [12,19], reducing falls [20,21,22], reversing frailty [23], and reducing the risk of suffering from it [6,24,25]. Additionally, improvements in cognitive function [26] and biochemical parameters such as the lipid profile and antioxidant function [27] and even reducing risk factors for multiple comorbidities [13] are also effects attributed to MCT. Furthermore, MCT has been shown to increase motivation for exercise participation in this population, even more when performed collectively [7,28].

Considering that the elderly population is increasing in number, and the proven potential of MCT to improve and maintain functional capacity, quality of life, and the overall health of this population group [13,29,30,31,32], it is relevant to know more about the state of this field of research to identify relevant topics in the field, new emerging lines of investigation, and relevant publications and authors. Thus, it is necessary to conduct a bibliometric review as a method to provide transparency, objectivity, and a complete overview of a research topic, in this case multicomponent exercise. This study is the first bibliometric analysis of MCT in the aged population, which seeks to provide an overview of the state of the art in this area through the application of the traditional laws of bibliometrics. The main objectives of this study were to (1) study if there exists an exponential growth in the number of annual publications on MCT in the aged population; (2) identify the most prolific and prominent co-authors and the relevant groups of co-authors; (3) highlight the countries that publish more on the topic; (4) identify the journals that publish the most on this topic; and (5) identify the most frequently used keywords and the subtopics they generate.

## 2. Materials and Methods

### 2.1. Design

This study is a descriptive bibliometric analysis of research on MCT in the aged population. The publications included in this scientific mapping were from the Web of Science Core Collection, which is considered the reference database for bibliometric studies [33,34,35] since it provides basic information on the authors, journals, affiliations, countries, citations, and other information of the indexed documents [36].

### 2.2. Data Search Strategy

The search terms included were (a) multicomponent training (and its synonyms and similar terms) and (b) aged population (and its synonyms and similar terms). The search was conducted using the following tags: TI (title search), AB (abstract search), and AK (author keywords). The full search vector is described in Table 1.

The following inclusion criteria were defined:

1. Being an original study or review;
2. Aged or older adult participants;
3. Addressing MCT;
4. Study involving humans.

All studies published up to 22 November 2023 were considered. The document selection process was independently performed by two authors (D.P.P. and J.C.V.), and in case of disagreement on the inclusion or non-inclusion of a document, a third author resolved the dispute (J.P.G.).

### 2.3. Data Analysis

The data cleaning process was carried out by eliminating duplicates in co-authors and keywords. The process of data analysis assessed (a) the stage of development of the research topic, through Price’s Law, which studies the degree of fit of the annual growth in the number of publications with respect to an exponential ratio [37]. (b) Lotka’s law was applied to find the most prolific authors. To verify the result estimated by Lotka’s law, a discrete count of co-authors was performed, and the coefficient of determination (R2) was calculated in Microsoft Excel. To identify prominent authors, (1) the most cited papers were identified by applying the Hirsch index (h-index, the set of “h” papers with “h” or more citations); (2) the number of most cited papers presented by each of the prolific authors was checked; and (3) prolific authors with at least one highly cited paper were considered prominent authors [38]. (c) Bradford’s law of concentration of scientific production was applied, identifying those with the most publications as the core journals on the topic [39,40]. (d) Zipf’s Law was applied to study the co-occurrence of keywords and to highlight the most used on this topic [41,42]. This result was verified through a discrete counting of author keywords and their co-occurrences, and the power law was adjusted by calculating R2 in Microsoft Excel.

Data were retrieved from the WoS database in .xslx and plain text format and subsequently analyzed in Microsoft Excel for Microsoft 365 MSO version 2206 (Washington, DC, USA) and VoSViewer software v. 1.6.18 (Leiden, The Netherlands).

## 3. Results

**Figure 1:**
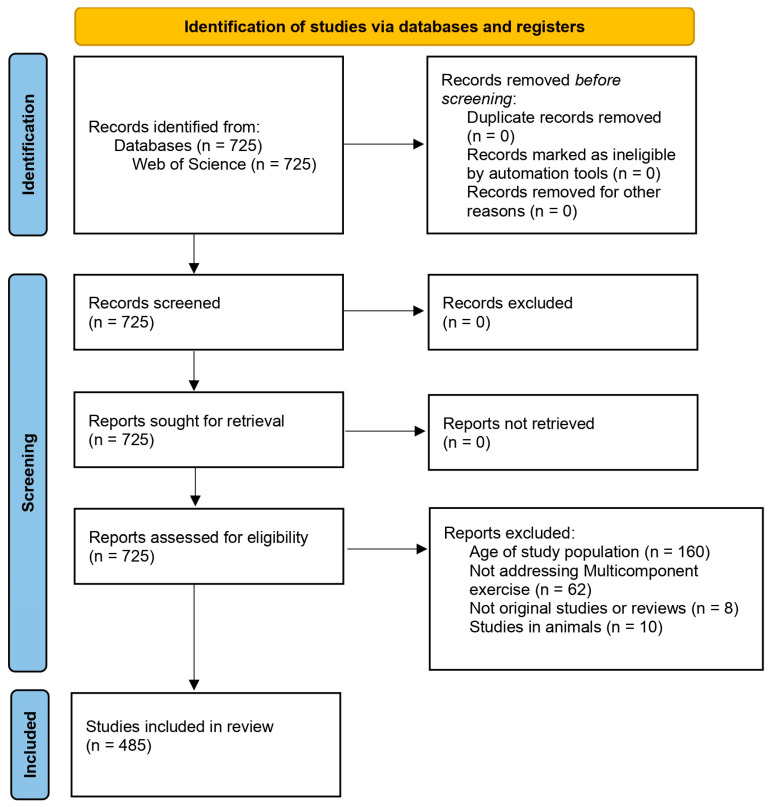
PRISMA 2020 flow diagram to describe the document selection process.

The flowchart in Figure 1 shows the document selection process. Finally, 485 documents were included.

### 3.1. Annual Publication Trends

**Figure 2:**
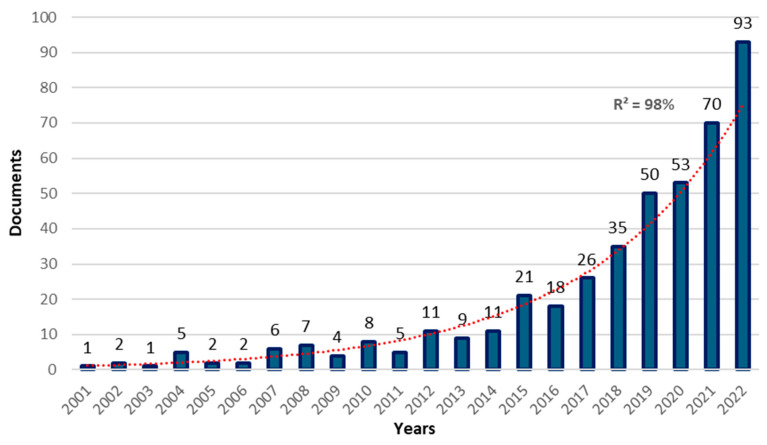
The trend in annual publications of multicomponent training.

From 2001, the year of the first publication, to 2010, 38 documents were published in this area. This number increased by six times (239 papers) in the following decade (2011–2020) and has not stopped growing. Between 2001 and 2022, the increasing trend in publications was evident with an R2 of 98%, in line with an exponential growth rate (Figure 2).

### 3.2. Categories

The documents were the result of investigations in different areas of knowledge, as it was found that the documents included in this review were related to 64 different thematic categories in the WoS. Table 2 shows the main thematic categories in which the papers were listed, including the publishers that contributed the most papers to each category. The top categories were geriatrics gerontology (181 papers), sports sciences (84 papers), and gerontology (65 papers).

### 3.3. Authors and Documents

**Figure 3:**
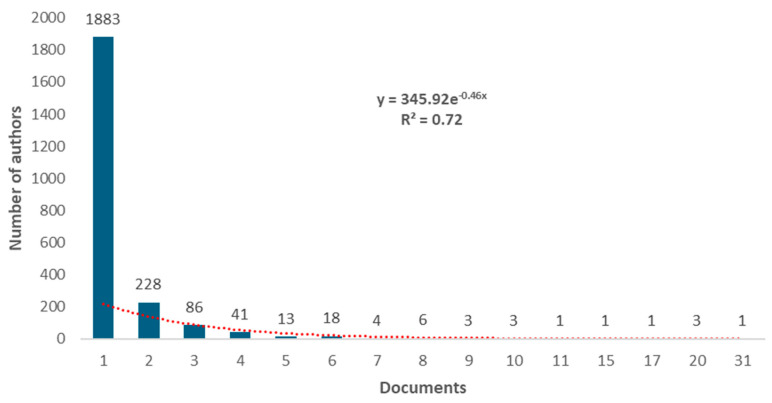
Histogram with the number of authors according to the number of published papers.

After eliminating duplicities and normalizing the names of the authors, 2292 different co-authors were found. It was estimated that the number of prolific authors should be equal to or less than 48 (square root of 2292). We found 54 authors with five or more papers and 41 authors with six or more papers; the latter were considered prolific authors. The range of papers per author was between 1 and 31 (Figure 3). Only one author had 31 documents on the topic, considered the most prominent, whereas 1883 authors limited their contribution to 1 document.

**Figure 4:**
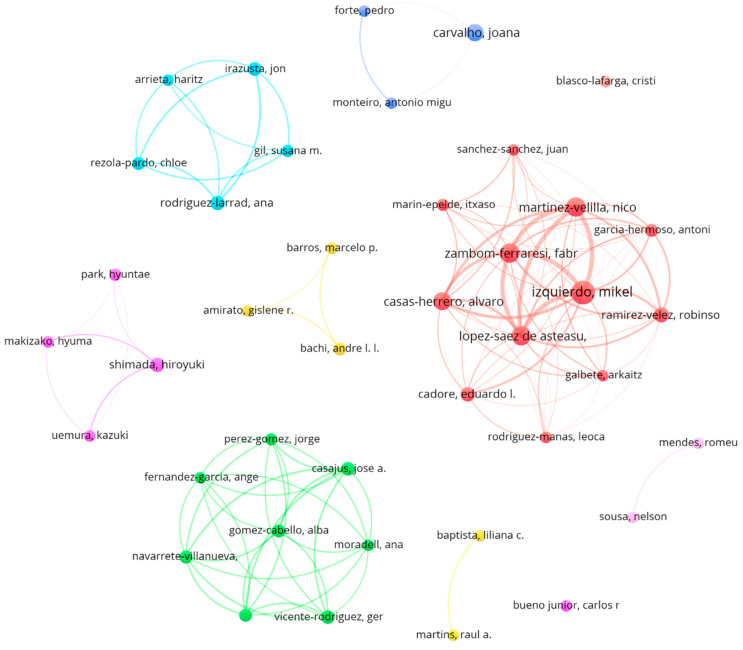
Graph showing the production networks formed by the prolific authors.

Figure 4 shows the prolific authors and the collaborative networks formed by them. A large production group (red cluster: 12 prolific authors) was found with the five most productive authors: M. Izquierdo (31 papers); M. López-Saez de Asteasu, N. Martínez-Velilla, and F. Zambom-Ferraresi (20 papers); and A. Casas-Herrero (17 papers). In terms of number of papers, J. Carvalho also stood out (17 papers), leading a small cluster together with P. Forte and A. Monteiro (6 papers).

**Figure 5:**
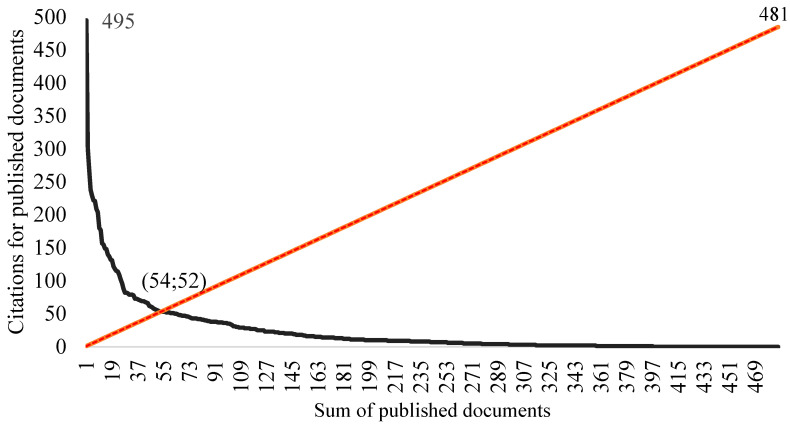
Graph representing the application of the h-index. Cut-off point: 52 documents and 54 citations.

When analyzing the number of times the documents were cited, it was found that the documents presented a range between 0 and 495 received citations. Figure 5 shows the h-index intercept, indicating that 52 papers had 54 or more citations. These 52 documents, with 54 citations or over, were considered the most cited on the topic (Table S1).

After analyzing the authors of the 52 most cited papers, 19 authors were identified as prominent authors. To be a prominent author, it was necessary to have published six or more papers and to present at least one paper among the most cited (with 54 or more citations). M. Izquierdo (31 papers, 1956 citations, 8 papers among the most cited); E. Cadore (10 papers, 1377 citations, 6 papers among the most cited); and L. Rodríguez-Manas (6 papers, 1676 citations, 6 papers among the most cited) stood out from the rest of the authors. These three authors belonged to the same production cluster (red cluster, Figure 4). Table 3 shows the prominent authors in the research area.

### 3.4. Countries

**Figure 6:**
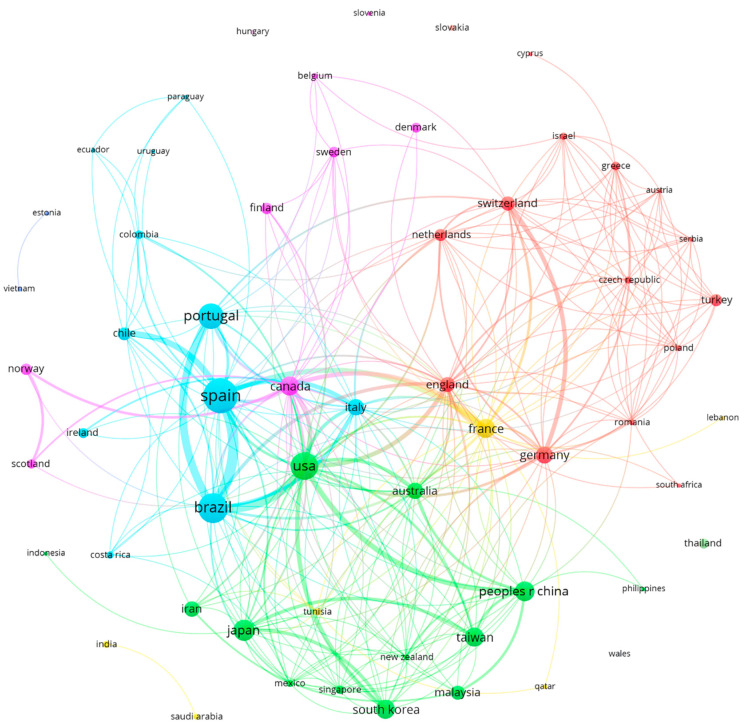
Inter-country production networks. Five major production clusters: red (14 countries), green (13 countries), blue (11 countries), pink (7 countries), and yellow (4 countries).

In the analysis of co-authorship by country, a total of 58 countries were found. Spain (98 documents, 3182 citations) was the country with the highest number of contributions. Other countries of note were Brazil (70 papers, 947 citations), USA (58 papers, 1881 citations), Portugal (49 papers, 588 citations), and Japan (33 papers, 901 citations). Figure 4 shows the 58 countries and the global production network formed by them. Five major production clusters were found: red (14 countries, with Germany, England, and Switzerland as the most productive countries); green (13 countries, with USA, Japan, and China among others); blue (11 countries, with very productive countries such as Spain, Brazil, and Portugal); pink (7 countries, including Canada, Finland, and Norway); and yellow (4 countries, with France as the main contributor) (Figure 6).

### 3.5. Journals

A total of 224 journals were found. The range of documents per journal was found to be between 1 and 23. Considering the number of documents, the journals were distributed into three zones: core (15 journals, 165 documents), Zone I (176 documents, 65 journals), and Zone II (144 documents, 144 journals). Table 4 shows Bradford’s zones and their indicators.

**Table 4:** Bradford’s zones.

Zone	Number of Documents on Thirds (%)		Journals (%)		Bradford Multipliers	Journals (Theoretical Series)	
CORE	165	34%	15	7%		15 × (n0)	15
ZONE I	176	36%	65	29%	4.3	15 × (n1)	49
ZONE II	144	30%	144	64%	2.2	15 × (n2)	161
TOTAL	485	100%	224	100%	3.3		225
						% Error	−0.4%

The journals that encompassed the publication core are shown in Table 5. Among them were the International Journal of Environmental Research and Public Health (23 papers), BMC Geriatrics (21 papers), and Aging Clinical and Experimental Research (15 papers).

**Table 5:** Core journals in multicomponent training publications.

Journal Name	Documents	Citations
International Journal of Environmental Research and Public Health	23	176
BMC Geriatrics	21	411
Aging Clinical and Experimental Research	15	321
Experimental Gerontology	14	311
Journal of Aging and Physical Activity	13	209
Archives of Gerontology and Geriatrics	11	208
Journal of the American Medical Directors Association	9	423
Medicine and Science in Sports and Exercise	9	1
Age and Ageing	8	171
Clinical Interventions in Aging	8	424
Nutrients	8	88
Frontiers in Aging Neuroscience	7	35
Geriatrics & Gerontology International	7	129
Medicine & Science in Sports & Exercise	6	3
Osteoporosis International	6	354

### 3.6. Keywords

**Figure 7:**
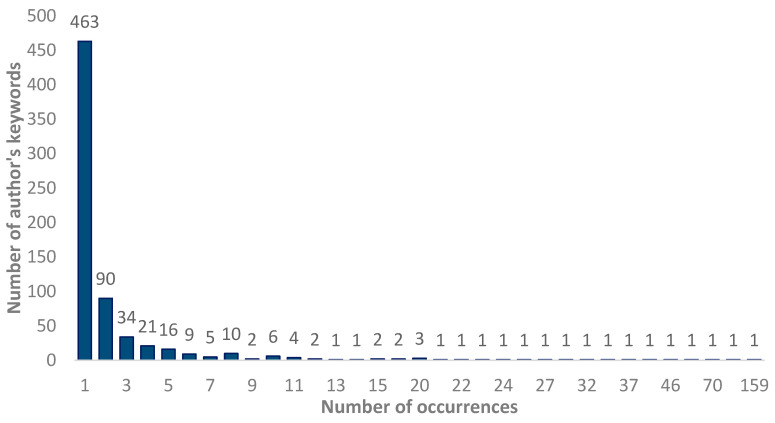
Relationship between the number of keywords and the number of keywords used by authors.

When analyzing author keywords, 821 terms were found. After the normalization process, 687 concepts were found. Applying Zipf’s law, it was estimated that the most important keywords should be the 26 most used (square root of 687) (Figure 7). Twenty-five keywords were found with 13 or more uses, and these were the most prominent in this subject matter. One keyword had 159 appearances, considered the most used, while 463 keywords appeared only one time on this research topic.

Table 6 shows the most used keywords and their frequency of use in this research area.

**Figure 8:**
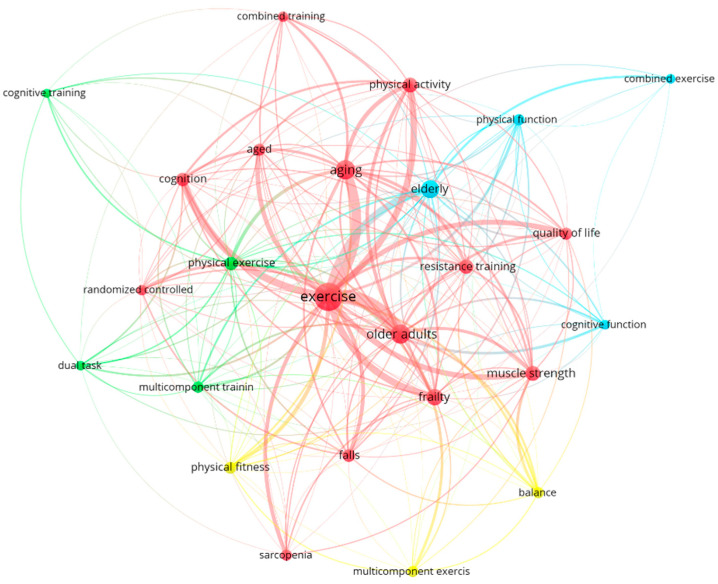
Author keywords’ co-occurrence graph. Four clusters of keywords were formed: red (strength, frailty, falls, and sarcopenia); green (cognitive training and dual-task exercise); blue (physical and cognitive function); and yellow (balance).

Figure 8 shows the co-occurrence analysis of these keywords. In this analysis, it was found that the keywords formed four clusters with the following colors and main themes: red (focused on strength, strength training, frailty, falls, and sarcopenia); green (focused on cognitive training and dual-task exercise); blue (focused on physical and cognitive function in the elderly); and yellow (focused on balance) (Figure 8).

## 4. Discussion

This is the first bibliometric analysis of the state of the art in MCT in the aged population. The present manuscript provides a systematic and quantitative analysis of the evolution of this research topic through all the documents found in WoS-indexed journals. The study of the 485 documents that met the inclusion criteria confirmed that research regarding MCT in the aged population is going through an exponential growth phase. Additionally, the present work provides information regarding the most relevant co-authors and works, the most prolific countries publishing on the topic, the journals most interested in this topic, and the frequent themes, identifying the most frequently used keywords.

From the year 2001, when the first item on the subject was published in a WoS-indexed journal, to 2022, the interest in this topic has increased, as evidenced by the exponential growth of the number of publications per year. In fact, PA in the older population has been a topic of growing interest from 2013 to 2021, as other bibliometric studies have shown [43]. Regarding PA and aging, an increase in the number of publications per year was reported from 1980 to 2014 [44]. Other investigations on the anti-aging effect of PA also reported an increase in the number of published items from 1991 to 2022 [45]. Unfortunately, there is no other bibliometric analysis about MCT in the elderly, but as the current research shows, the issue of PA in the aged population is a subject of growing interest. One of the reasons for this increased interest in MCT in the elderly may be due to the positive effects that this type of training has been shown to have [14,15,16,17], which encourages more research to be carried out in this area. At the same time, the growing number of elderly people [1] may be causing a boom in the use of this sector of the population as a study population since there would be a greater number of potential benefits derived from research involving this population.

The top three WoS categories in which journals published more on the subject were geriatrics gerontology, sports sciences, and gerontology. In terms of journals, 224 was the total number of published journals in this area. Notably, 15 of these journals formed the core journals on the topic. Among them, the top three most prolific were the International Journal of Environmental Research and Public Health, BMC Geriatrics, and Aging Clinical and Experimental Research. Other bibliometric studies regarding a great variety of topics have also found the International Journal of Environmental Research and Public Health as one of the journals with more publications; this may be due to the interdisciplinary approach of this journal [35,46,47].

The analysis of co-authors identified 41 co-authors with six or more publications on the subject, who were defined as prolific. At the same time, 19 prominent co-authors were found. The most highlighted co-author in both categories was Mikel Izquierdo, who had the highest number of publications and citations on this topic, and who in turn led the most outstanding cluster of co-authors on this topic, as revealed by the analysis of collaborations between co-authors. This cluster was mainly formed by authors from the Universidad Pública de Navarra and the Navarrabiomed Biomedical Research Center and included the top five authors with the highest number of publications on this topic and the top nine authors with the highest number of citations. This is because this collaborative group has published a large number of papers on this topic, and their works have had a significant impact and, consequently, have received a great number of citations. In fact, eight of the most cited papers on the subject were co-authored by members of this cluster [19,48,49,50,51,52,53,54].

The existence of such an outstanding group of collaboration in the field, as is the case with the aforementioned researchers, together with another important cluster (green cluster) formed entirely by authors affiliated with Spanish institutions, justifies the claim that Spain is the largest producer of scientific research in the field of MCT for the aged population and leads the most prominent inter-country collaboration group.

Finally, the most frequently used keywords and the frequency with which they were used together were analyzed to study the most common topics in this research field. Four clusters of keywords were found. One centered on strength training [55], sarcopenia [51], quality of life [56], and falls [57]. Another focused on cognitive training [58] and dual-task exercise [59]. Another revolved around cognitive [60] and physical functioning [61], and the final one focused on balance [6].

From this analysis, we can highlight that resistance training is one of the most frequently used forms of training in MCT [29,62]. Great importance is given to sarcopenia and falls in this population, which is linked to the balance cluster. This indicates that balance is one of the most sought-after targets for improvement in MCT research [19,29]. Additionally, cognitive training and dual-task exercise also stand out with some relevant publications that focus on the development and maintenance of cognitive and functional capacity in the aged population [63,64]. Other studies found that, in research about PA in older adults, the main groups of keywords were related to sarcopenia, cognition, frailty, mental health, and rehabilitation [44]. We can see that, in comparison with the present study, the keywords related to the different types of training are not so important, but nevertheless, the themes of cognition and falls are common. The analysis of the keywords allows us to identify some themes that have not been developed so far on this research topic yet and that may be of interest in the future. This is the case for mental health since no term related to it was among the most used keywords; this can be a potential field of study for future research given the proven effect of exercise on its improvement [65].

### 4.1. Practical Implications

The present bibliometric analysis offers a broad analysis of research in MCT and the aged population. The information here provided allows researchers to identify the journals that publish the most in this field, the topics that raised the greatest interest, the most prolific and prominent authors, and the referential works in this field of study. This information will help future researchers in the field to plan and orient their investigations, as well as to identify new lines of research.

### 4.2. Limitations

This work was only based on publications indexed in the WoS, which is a source of bias and constitutes the main limitation of this investigation. Additionally, although every effort was made to include all terms related to the subject matter in the search vector, some words linked to the topic may have been left out. This may have limited the number of documents included in the analysis.

## 5. Conclusions

This bibliometric analysis identified a total of 485 documents on MCT in the aged population. An exponential growth in the number of publications on the topic was found in the studied period from 2001 to 2022. Geriatrics gerontology, sports sciences, and gerontology were the three WoS categories with the highest number of publications on the subject.

The most prolific co-authors were Mikel Izquierdo (31 documents) and Mikel Lopez-Sáez de Asteasu, Nicolas Martinez-Velilla, and Fabricio Zambom-Ferraresi (with 20 documents each). Additionally, Eduardo Lusa Cadore, Leocadio Rodriguez-Manas, and Mikel Izquierdo stood out from the rest of the prominent co-authors. All of them, together with 13 other co-authors affiliated with Spanish institutions, belonged to a collaborative network that excelled in this area. Spain was identified as the most productive nation on the topic. Alongside Spain, Germany, USA, Canada, and France led the top five clusters of collaboration between countries.

Regarding journals, 15 of them with six or more publications each were identified as the core journals. The top three most prolific were the International Journal of Environmental Research and Public Health, BMC Geriatrics, and Aging Clinical and Experimental Research.

The most frequently addressed themes in this research area were focused on strength, sarcopenia, quality of life, falls, and balance. Cognitive and physical functioning, cognitive training, and dual-task exercise were other relevant subjects more related to functional and cognitive capacity, on which co-authors also published frequently.

## Figures and Tables

**Table 1:** Search vector.

TI = ((“combined training” OR “combined exercise” OR “multi-component training” OR “multi-component exercise” OR “multicomponent training” OR “multicomponent exercise”) AND (“aged” OR “old people” OR “older people” OR “older adults” OR “old adults” OR “elderly” OR “senior” OR “geriatric” OR “frail”)) OR (AB = ((“combined training” OR “combined exercise” OR “multi-component training” OR “multi-component exercise” OR “multicomponent training” OR “multicomponent exercise”) AND (“aged” OR “old people” OR “older people” OR “older adults” OR “old adults” OR “elderly” OR “senior” OR “geriatric” OR “frail”))) OR (AK = ((“combined training” OR “combined exercise” OR “multi-component training” OR “multi-component exercise” OR “multicomponent training” OR “multicomponent exercise”) AND (“aged” OR “old people” OR “older people” OR “older adults” OR “old adults” OR “elderly” OR “senior” OR “geriatric” OR “frail”)))

**Table 2:** Main thematic categories to which the documents were related.

	Web of Sciences Categories	Number of Documents	Prolific Publishers	Number of Documents
1	Geriatrics Gerontology	181	Springer Nature	91
2	Sports Sciences	84	Elsevier	68
3	Gerontology	65	MDPI	56
4	Public Environmental Occupational Health	42	Wiley	28
5	Medicine General Internal	34	Frontiers Media Sa	26

**Table 3:** Prominent authors on this topic (in no particular order).

Author Name	Total Documents	Citations	Papers among Most Cited	Orcid
Izquierdo, M.	31	1956	8	0000-0002-1506-4272
Cadore, E.	10	1377	6	0000-0003-4397-9485
Rodriguez-Manas, L.	6	1676	6	0000-0002-6551-1333
Lopez-Saez de Asteasu, M.	20	710	3	0000-0002-4111-5045
Martínez-Velilla, N.	20	654	2	0000-0001-9576-9960
Zambom-Ferraresi, F.	20	803	3	0000-0002-8377-9827
Casas-Herrero, A.	17	990	4	0000-0001-8430-8368
Carvalho, J.	15	263	2	0000-0001-6500-7543
Ramírez-Velez, R.	11	383	1	0000-0003-3075-6960
Shimada, H.	10	360	2	0000-0001-8111-6440
Casajús, J.	9	271	1	0000-0002-7215-6931
Vicente-Rodríguez, G.	9	274	1	0000-0002-4303-4097
Ara, I.	8	262	1	0000-0002-2854-6684
Garcia-Hermoso, A.	8	334	1	0000-0002-1397-7182
Gomez-Cabello, A.	8	271	1	0000-0001-6492-2512
Park, H.	7	288	1	0000-0002-1976-0005
Galbete, A.	6	369	1	0000-0001-5622-5418
Makizako, H.	6	338	2	0000-0001-9898-675X
Uemura, K.	6	229	1	0000-0003-1101-3424

**Table 6:** More frequently used concepts on this topic.

Position	Keyword	Appearances
1	Exercise	159
2	Older adults	72
3	Aging	70
4	Elderly	56
5	Frailty	46
6	Muscle strength	39
7	Physical activity	37
8	Resistance training	33
9	Physical exercise	32
10	Cognition	31
11	Falls	27
12	Quality of life	25
13	Aged	24
14	Physical fitness	23
15	Multicomponent exercise	22
16	Physical function	21
17	Balance	20
18	Multicomponent training	20
19	Sarcopenia	20
20	Combined training	19
21	Randomized controlled trial	19
22	Cognitive function	15
23	Dual task	15
24	Combined exercise	14
25	Cognitive training	13

## Data Availability

Data are contained within the article and Supplementary Materials.

## Supplementary Materials

The following supporting information can be downloaded at: https://www.mdpi.com/article/10.3390/healthcare12151493/s1, Table S1: Most cited documents on the topic.
