# Supplementary material for: Trends in Multicomponent Training Research in the Aged Population: A Bibliometric Analysis
Source: Healthcare (Basel). 2024 Jul 27;12(15):1493. doi: 10.3390/healthcare12151493 (PMC11311504; doi:10.3390/healthcare12151493)
Supplement: Supplementary file 1 [file healthcare-12-01493-s001.zip › healthcare-3110441-supplementary.pdf]

Table S1. Most cited documents in the topic.

AUTHORS	TITLE	JOURNAL ABREVIATION
Cadore, EL; Rodríguez-Mañas, L; Sinclair, A; Izquierdo, M	Effects of Different Exercise Interventions on Risk of Falls, Gait Ability, and Balance in Physically Frail Older Adults: A Systematic Review	REJUV RES
Tarazona-Santabalbina, FJ; Gómez-Cabrera, MC; Pérez-Ros, P; Martínez-Arnau, FM; Cabo, H; Tsaparas, K; Salvador-Pascual, A; Rodriguez-Mañas, L; Viña, J	A Multicomponent Exercise Intervention that Reverses Frailty and Improves Cognition, Emotion, and Social Networking in the Community-Dwelling Frail Elderly: A Randomized Clinical Trial	J AM MED DIR ASSOC
Izquierdo, M; Merchant, RA; Morley, JE; Anker, SD; Aprahamian, I; Arai, H; Aubertin-Leheudre, M; Bernabei, R; Cadore, EL; Cesari, M; Chen, LK; Barreto, PD; Duque, G; Ferrucci, L; Fielding, RA; García-Hermoso, A; Gutiérrez-Robledo, LM; Harridge, SDR; Kirk, B; Kritchevsky, S; Landi, F; Lazarus, N; Martin, FC; Marzetti, E; Pahor, M; Ramírez-Vélez, R; Rodriguez-Mañas, L; Rolland, Y; Ruiz, JG; Theou, O; Villareal, DT; Waters, DL; Won, CW; Woo, J; Vellas, B; Singh, MF	International Exercise Recommendations in Older Adults (ICFSR): Expert Consensus Guidelines	J NUTR HEALTH AGING
Martínez-Velilla, N; Casas-Herrero, A; Zambom-Ferraresi, F; de Asteasu, MLS; Lucia, A; Galbete, A; García-Baztán, A; Alonso-Renedo, J; González-Glaría, B; Gonzalo-Lázaro, M; Iráizoz, IA; Gutiérrez-Valencia, M; Rodríguez-Mañas, L; Izquierdo, M	Effect of Exercise Intervention on Functional Decline in Very Elderly Patients During Acute Hospitalization A Randomized Clinical Trial	JAMA INTERN MED
Gómez-Cabello, A; Ara, I; González-Agüero, A; Casajús, JA; Vicente-Rodríguaz, G	Effects of Training on Bone Mass in Older Adults	SPORTS MED
Barha, CK; Davis, JC; Falck, RS; Nagamatsu, LS; Liu-Ambrose, T	Sex differences in exercise efficacy to improve cognition: A systematic review and meta-analysis of randomized controlled trials in older humans	FRONT NEUROENDOCRIN
Cadore, EL; Moneo, ABB; Mensat, MM; Muñoz, AR; Casas-Herrero, A; Rodriguez-Mañas, L; Izquierdo, M	Positive effects of resistance training in frail elderly patients with dementia after long-term physical restraint	AGE

Daniels, R; van Rossum, E; de Witte, L; Kempen, GIJM; van den Heuvel, W	Interventions to prevent disability in frail community-dwelling elderly: a systematic review	BMC HEALTH SERV RES
Davidson, LE; Hudson, R; Kilpatrick, K; Kuk, JL; McMillan, K; Janiszewski, PM; Lee, S; Lam, M; Ross, R	Effects of Exercise Modality on Insulin Resistance and Functional Limitation in Older Adults A Randomized Controlled Trial	ARCH INTERN MED
Paw, MJMCA; van Uffelen, JGZ; Riphagen, I; van Mechelen, W	The functional effects of physical exercise training in frail older people - A systematic review	SPORTS MED
de Labra, C; Guimaraes-Pinheiro, C; Maseda, A; Lorenzo, T; Millán-Calenti, JC	Effects of physical exercise interventions in frail older adults: a systematic review of randomized controlled trials	BMC GERIATR
Suzuki, T; Shimada, H; Makizako, H; Doi, T; Yoshida, D; Tsutsumimoto, K; Anan, Y; Uemura, K; Lee, S; Park, H	Effects of multicomponent exercise on cognitive function in older adults with amnestic mild cognitive impairment: a randomized controlled trial	BMC NEUROL
Cadore, EL; Casas-Herrero, A; Zambom-Ferraresi, F; Idoate, F; Millor, N; Gómez, M; Rodriguez-Mañas, L; Izquierdo, M	Multicomponent exercises including muscle power training enhance muscle mass, power output, and functional outcomes in institutionalized frail nonagenarians	AGE
Mathus-Vliegen, EMH; Basdevant, A; Finer, N; Hainer, V; Hauner, H; Micic, D; Maislos, M; Roman, G; Schutz, Y; Tsigos, C; Toplak, H; Yumuk, V; Zahorska-Markiewicz, B	Prevalence, Pathophysiology, Health Consequences and Treatment Options of Obesity in the Elderly: A Guideline	OBESITY FACTS
Mathus-Vliegen, EMH	Obesity and the Elderly	J CLIN GASTROENTEROL
Giangregorio, LM; Papaioannou, A; MacIntyre, NJ; Ashe, MC; Heinonen, A; Shipp, K; Wark, J; McGill, S; Keller, H; Jain, R; Laprade, J; Cheung, AM	Too Fit To Fracture: exercise recommendations for individuals with osteoporosis or osteoporotic vertebral fracture	OSTEOPOROSIS INT
Karinkanta, S; Heinonen, A; Sievänen, H; Uusi-Rasi, K; Pasanen, M; Ojala, K; Fogelholm, M; Kannus, P	A multi-component exercise regimen to prevent functional decline and bone fragility in home-dwelling elderly women:: randomized, controlled trial	OSTEOPOROSIS INT
Eggenberger, P; Schumacher, V; Angst, M; Theill, N; de Bruin, ED	Does multicomponent physical exercise with simultaneous cognitive training boost cognitive performance in older adults? A 6-month randomized controlled trial with a 1-year follow-up	CLIN INTERV AGING
Freiberger, E; Häberle, L; Spirduso, WW; Zijlstra, GAR	Long-Term Effects of Three Multicomponent Exercise Interventions on Physical Performance and Fall-Related Psychological Outcomes in Community-Dwelling Older Adults: A Randomized Controlled Trial	J AM GERIATR SOC
de Asteasu, MLS; Martínez-Velilla, N; Zambom-Ferraresi, F; Casas-Herrero, A; Izquierdo, M	Role of physical exercise on cognitive function in healthy older adults: A systematic review of randomized clinical trials	AGEING RES REV

Bibas, L; Levi, M; Bendayan, M; Mullie, L; Forman, DE; Afilalo, J	Therapeutic Interventions for Frail Elderly Patients: Part I. Published Randomized Trials	PROG CARDIOVASC DIS
Mador, MJ; Bozkanat, E; Aggarwal, A; Shaffer, M; Kufel, TJ	Endurance and strength training in patients with COPD	CHEST
Karinkanta, S; Piirtola, M; Sievänen, H; Uusi-Rasi, K; Kannus, P	Physical therapy approaches to reduce fall and fracture risk among older adults	NAT REV ENDOCRINOL
Forte, R; Boreham, CAG; Leite, JC; De Vito, G; Brennan, L; Gibney, ER; Pesce, C	Enhancing cognitive functioning in the elderly: multicomponent vs resistance training	CLIN INTERV AGING
Carvalho, MJ; Marques, E; Mota, J	Training and Detraining Effects on Functional Fitness after a Multicomponent Training in Older Women	GERONTOLOGY
Bouaziz, W; Lang, PO; Schmitt, E; Kaltenbach, G; Geny, B; Vogel, T	Health benefits of multicomponent training programmes in seniors: a systematic review	INT J CLIN PRACT
Chen, FT; Etnier, JL; Chan, KH; Chiu, PK; Hung, TM; Chang, YK	Effects of Exercise Training Interventions on Executive Function in Older Adults: A Systematic Review and Meta-Analysis	SPORTS MED
Barreto, PD; Rolland, Y; Vellas, B; Maltais, M	Association of Long-term Exercise Training With Risk of Falls, Fractures, Hospitalizations, and Mortality in Older Adults A Systematic Review and Meta-analysis	JAMA INTERN MED
Aguirre, LE; Villareal, DT	Physical Exercise as Therapy for Frailty	NESTLE NUTR WORKS SE
Desjardins-Crépeau, L; Berryman, N; Fraser, SA; Vu, TTM; Kergoat, MJ; Li, KZH; Bosquet, L; Bherer, L	Effects of combined physical and cognitive training on fitness and neuropsychological outcomes in healthy older adults	CLIN INTERV AGING
Levin, O; Netz, Y; Ziv, G	The beneficial effects of different types of exercise interventions on motor and cognitive functions in older age: a systematic review	EUR REV AGING PHYS A
Villareal, DT; Smith, GI; Sinacore, DR; Shah, K; Mittendorfer, B	Regular Multicomponent Exercise Increases Physical Fitness and Muscle Protein Anabolism in Frail, Obese, Older Adults	OBESITY
Toraman, NF; Ayceman, N	Effects of six weeks of detraining on retention of functional fitness of old people after nine weeks of multicomponent training	BRIT J SPORT MED
Low, DC; Walsh, GS; Arkesteijn, M	Effectiveness of Exercise Interventions to Improve Postural Control in Older Adults: A Systematic Review and Meta-Analyses of Centre of Pressure Measurements	SPORTS MED
Zhu, LY; Chan, R; Kwok, T; Cheng, KCC; Ha, A; Woo, J	Effects of exercise and nutrition supplementation in community-dwelling older Chinese people with sarcopenia: a randomized controlled trial	AGE AGEING
Tseng, CN; Gau, BS; Lou, MF	The Effectiveness of Exercise on Improving Cognitive Function in Older People: A Systematic Review	J NURS RES

Suzuki, T; Shimada, H; Makizako, H; Doi, T; Yoshida, D; Ito, K; Shimokata, H; Washimi, Y; Endo, H; Kato, T	A Randomized Controlled Trial of Multicomponent Exercise in Older Adults with Mild Cognitive Impairment	PLOS ONE
Toraman, NF; Erman, A; Agyar, E	Effects of multicomponent training on functional fitness in older adults	J AGING PHYS ACTIV
Hollings, M; Mavros, Y; Freeston, J; Singh, MF	The effect of progressive resistance training on aerobic fitness and strength in adults with coronary heart disease: A systematic review and meta-analysis of randomised controlled trials	EUR J PREV CARDIOL
Rand, D; Eng, JJ; Liu-Ambrose, T; Tawashy, AE	Feasibility of a 6-Month Exercise and Recreation Program to Improve Executive Functioning and Memory in Individuals With Chronic Stroke	NEUROREHAB NEURAL RE
Helbostad, JL; Sletvold, O; Moe-Nilssen, R	Effects of home exercises and group training on functional abilities in home-dwelling older persons with mobility and balance problems. A randomized study	AGING CLIN EXP RES
Helbostad, JL; Sletvold, O; Moe-Nilssen, R	Home training with and without additional group training in physically frail old people living at home: effect on health-related quality of life and ambulation	CLIN REHABIL
Castrogiovanni, P; Trovato, FM; Szychlinska, MA; Nsir, H; Imbesi, R; Musumeci, G	The importance of physical activity in osteoporosis. From the molecular pathways to the clinical evidence	HISTOL HISTOPATHOL
Haeger, A; Costa, AS; Schulz, JB; Reetz, K	Cerebral changes improved by physical activity during cognitive decline: A systematic review on MRI studies	NEUROIMAGE-CLIN
Cadore, EL; de Asteasu, MLS; Izquierdo, M	Multicomponent exercise and the hallmarks of frailty: Considerations on cognitive impairment and acute hospitalization	EXP GERONTOL
Damirchi, A; Hosseini, F; Babaei, P	Mental Training Enhances Cognitive Function and BDNF More Than Either Physical or Combined Training in Elderly Women With MCI: A Small-Scale Study	AM J ALZHEIMERS DIS
VanSwearingen, JM; Perera, S; Brach, JS; Wert, D; Studenski, SA	Impact of Exercise to Improve Gait Efficiency on Activity and Participation in Older Adults With Mobility Limitations: A Randomized Controlled Trial	PHYS THER
Zhuang, J; Huang, L; Wu, YQ; Zhang, YX	The effectiveness of a combined exercise intervention on physical fitness factors related to falls in community-dwelling older adults	CLIN INTERV AGING
Timonen, L; Rantanen, T; Ryynänen, OP; Taimela, S; Timonen, TE; Sulkava, R	A randomized controlled trial of rehabilitation after hospitalization in frail older women:: effects on strength, balance and mobility	SCAND J MED SCI SPOR
Toraman, NF	Short term and long term detraining: is there any difference between young-old and old people?	BRIT J SPORT MED
Marques, E; Carvalho, J; Soares, JMC; Marques, F; Mota, J	Effects of resistance and multicomponent exercise on lipid profiles of older women	MATURITAS
Cadore, EL; Izquierdo, M	Exercise interventions in polypathological aging patients that coexist with diabetes mellitus: improving functional status and quality of life	AGE
